# Hypoxia- and radiation-induced overexpression of Smac by an adenoviral vector and its effects on cell cycle and apoptosis in MDA-MB-231 human breast cancer cells

**DOI:** 10.3892/etm.2013.1351

**Published:** 2013-10-16

**Authors:** WEI-WU LIU, YANG LIU, SHUO LIANG, JIA-HUI WU, ZHI-CHENG WANG, SHOU-LIANG GONG

**Affiliations:** 1 Key Laboratory of Radiobiology, Ministry of Health, School of Public Health, Jilin University, Changchun, Jilin 130021, P.R. China; 2 Department of Radiology, Second Hospital, Jilin University, Changchun, Jilin 130041, P.R. China

**Keywords:** hypoxia, radiation, second mitochondria-derived activator of caspase, breast cancer, apoptosis

## Abstract

A conditionally replicative adenoviral (CRAd) vector, designated as CRAd.pEgr-1-Smac, that promotes the overexpression of second mitochondria-derived activator of caspase (Smac) when stimulated by hypoxia and radiation was constructed. MDA-MB-231 cells were transfected with CRAd.pEgr-1-Smac and treated with 4-Gy X-rays. The hypoxic status in cancer cells was mimicked with the chemical reagent CoCl_2_. Smac protein expression was measured by a western blotting assay and cell proliferation was detected with the MTT assay. The cell cycle progression and apoptotic percentage were measured by flow cytometry with PI and Annexin V-FITC staining kits, respectively, following the irradiation of the transfected cells with 4-Gy X-rays. The results showed that CRAd.pEgr-1-Smac was able to increase the Smac protein expression induced by hypoxia and radiation, inhibit cell proliferation and promote apoptosis. Therefore, this method of gene-radiotherapy is indicated to be an ideal strategy for the treatment of breast cancer.

## Introduction

Breast cancer accounts for 30% of primary malignant tumors in women (1). Radiotherapy is an important method for the clinical treatment of breast cancer, but its curative effect is often affected by damage to the surrounding normal tissues and tumor radiation tolerance, so radiotherapy alone has certain limitations (2). Gene-radiotherapy, as a new therapy that combines gene therapy and radiation therapy, has attracted much interest and has broad application prospects (3,4). The basic principle of gene-radiotherapy is the use of the radiation-induced characteristics of early growth response-1 (Egr-1) to increase the expression of a target gene following radiation and thereby enhance the treatment effect. Egr-1, containing the six serum response elements of CArG [CC (A + T-rich) 6GG], is a key component of radiation-activated expression. Numerous studies have observed that if the Egr-1 promoter gene is placed upstream of TNF-α, IFN-γ, endostatin and TRAIL genes, it promotes the expression of these genes by radiation induction (5–7). In the present study, the application of the radiotherapy-induced Egr-1 promoter gene is considered.

The target gene of tumor gene-radiotherapy may be a pro-apoptotic, cytokine or suicide gene (7–9). Ionizing radiation is able to induce the apoptosis and cell cycle arrest of tumor cells, and the failure to repair DNA damage following cell cycle arrest causes cell apoptosis (10). Therefore, second mitochondria-derived activator of caspase (Smac) was used as the target gene in the current study. Smac is localized in the mitochondria and released into the cytoplasm, triggering a cascade reaction of the caspase family through a variety of pathways, and promoting apoptosis. Smac is expressed in a variety of tumors, and is closely associated with the occurrence and development of various tumors (11). The overexpression of the Smac gene may promote the apoptosis of tumor cells and enhance the sensitivity of the cells to chemotherapy and radiotherapy. A previous study has shown that overexpression of the Smac gene may cause cancer cells to become more sensitive to apoptotic stimuli. In particular, a short amino acid sequence, which is separated from the N-terminus of the Smac protein, also reacts with XIAP and may kill tumor cells overexpressing IAPs (12,13). The purpose of the current study was to investigate the dual effects of apoptosis induced by ionizing radiation and the Smac gene.

Egr-1 may be activated by radiation to deliver gene therapy, but often the hypoxic microenvironment in solid tumors markedly reduces the effect of the Egr-1 promoter. Overcoming solid tumor hypoxia (leading to radiation tolerance) is a key challenge in the treatment of tumors. The core sequence of hypoxia response elements (HREs), 5′-(A/G)CGT(G/C)(G/C)-3′, has clear hypoxia-inducible characteristics (14–16). In addition, the use of specific replication with the conditionally replicative adenovirus (CRAd) in tumor cells is able to greatly increase the copy number and cause the high level expression of therapeutic genes (17). The conditionally replicative adenovirus mediated by HREs may achieve increased gene expression under hypoxic conditions and overcome the low efficiency of radiotherapy caused by the hypoxic environment.

Therefore, in the present study, HRE and Egr-1 were used to construct a CRAd vector to mediate the expression of the Smac gene when induced by the dual stimuli of hypoxia and radiation. The effects of the vector on the proliferation, cell cycle and apoptosis of MDA-MB-231 human breast cancer cells were then observed. This exploration of the gene-radiotherapy effect was conducted in order to provide new insight for the clinical radiotherapy of breast cancer.

## Materials and methods

### Cell lines and culture

MDA-MB-231 human breast cancer cells were purchased from the Shanghai Institute of Cell Biology, Chinese Academy of Science (Shanghai, China). The cells were cultured at 37°C with 5% CO_2_, using L15 medium containing 10% fetal bovine serum, 100 U/ml penicillin and streptomycin (Gibco BRL, Carlsbad, CA, USA). HEK293 human embryonic kidney cells were maintained by the Institute of Biochemistry and Cell Biology, Chinese Academy of Science (Shanghai, China). The cells were cultured under the same conditions as were used for the MDA-MB-231 cells, with a high sugar DMEM medium containing 10% fetal bovine serum, 100 U/ml penicillin and 100 U/ml streptomycin (Sigma, St. Louis, MO, USA).

### CRAd

**Figure 1:**
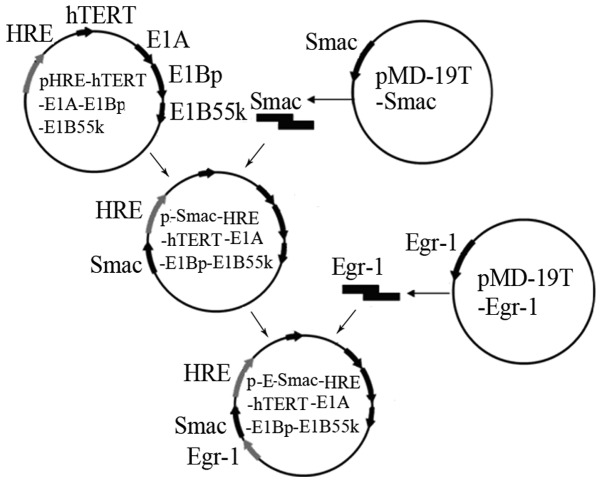
Construction of recombinant plasmid pShuttle-Egr-1-Smac-HRE-hTERT-E1A-E1Bp-E1B55K. Smac, second mitochondria-derived activator of caspase; Egr-1, early growth response-1; HRE, hypoxia response element.

A shuttle vector, pShuttle-Egr-1-Smac-HRE-hTERT-E1A-E1Bp-E1B55K was constructed. It was activated by hypoxia and radiation, resulting in the overexpression of Smac. The shuttle vector was transferred into BJ5183 (AdEasy-1 +) by electrotransformation, where it underwent homologous recombination with pAdEasy-1 to form the recombinant adenovirus plasmid. The plasmid was then transfected into HEK293 cells using the Lipofectamine 2000 regeants reagents (Invitrogen, Carlsbad, CA, USA). After packing, the CRAd was named CRAd.pEgr-1-Smac. The empty virus CRAd.p served as the control. After 3–5 generations of amplification, the virus was collected, the virus titer was confirmed by determining the 50% tissue culture infection dose (TCID_50_) and the virus was stored below −70°C. The establishment process of pShuttle-Egr-1-Smac-HRE-hTERT-E1A-E1Bp-E1B55K is shown in Fig. 1.

### Cell transfection, hypoxia and X-ray irradiation

The MDA-MB-231 cells were divided in six groups: normal control, CRAd.pEgr-1-Smac, hypoxia (H), empty virus (CRAd.p), CRAd.pEgr-1-Smac + H and CRAd.p + H. Briefly, the cells were seeded in 6-, 24- or 96-well culture plates. As the cells reached 80–90% confluence, they were infected with the adenovirus at a MOI [multiplicity of infection (virus/cell)] of five for 24 h according to the literature method (18). CoCl_2_ (Sigma) was added at a final concentration of 150 μmol/l for 24 h to simulate hypoxia (19). The X-ray irradiation was performed using an X-ray machine (X.S.S.250FZ; Guiyang Medical Instrument, Guiyang, China) under the following conditions: 200 kV, 10 mA, 0.5 mm Cu filter and 1.0 mm Al filter, a target skin distance of 50 cm, and 0 or 4 Gy of radiation at a dose rate of 0.287 Gy/min. The dose and dose rate options were in accordance with studies by the United Nations Scientific Committee on Atomic Radiation (UNSCAR) in 1986 and our previous work (7,20).

### Detection of the Smac protein

The MDA-MB-231 cells were seeded at a density of 1×10^6^ cells in each well of 6-well culture plates, followed by the treatment described in the preceding section. The cells were collected 24 h after irradiation and lysed using a lysis buffer [10 mmol/l Tris-HCl, pH 7.4; 1 mmol/l EDTA, pH 8.0; 0.1 mol/l NaCl; 1 μg/ml aprotinin; 100 μg/ml phenylmethanesulfonyl fluoride (PMSF)]. The total protein was extracted, quantified using a Coomassie blue protein quantification kit (Nanjing Jiancheng Biological Institute, Nanjing, China) and separated by 12% SDS-PAGE (loading: 50 μg). The protein was electroblotted onto a nitrocellulose membrane. Then, the membrane was incubated consecutively in 5% nonfat milk for blocking for 1 h and with anti-GAPDH or anti-Smac antibodies (Santa Cruz Biotechnology, Inc., Santa Cruz, CA, USA) overnight at 4°C. The membrane was then washed in TBST buffer and incubated with horseradish peroxidase-conjugated secondary antibody at 37°C for 1 h (Thermo Fisher Scientific Inc., Lake Barrington, IL, USA). The western blotting luminal reagent (Santa Cruz Biotechnology, Inc.) was used to develop the blots, followed by the photographic capturing of images for analysis.

### Detection of cell proliferation

The 3-(4,5-dimethylthiazol-2-yl) -2,5-diphenyltetrazolium bromide (MTT) method was used to detect cell proliferation. Briefly, the MDA-MB-231 cells were seeded at a density of 2×10^4^ cells per well in a 96-well culture plate, with six replicates for each group, and treated as described previously. At 12, 24 and 48 h after irradiation, 10 μl MTT (5 mg/ml; Sigma) was added for 4 h. The supernatants were discarded and 100 μl dimethylsulfoxide (DMSO; Sigma) was added to dissolve the crystals. The optical density (OD) value at 570 nm was measured using a microplate reader (Bio-Rad, Hercules, CA, USA) (7). The experiment was repeated three times.

### Flow cytometric analysis of the cell cycle and apoptosis

The cell cycle and apoptosis were analyzed by flow cytometry (FCM; Becton-Dickinson, Franklin Lakes, NJ, USA) with PI single dye (Sigma) or PI + Annexin V-FITC double staining (Nanjing KeyGEN Biotech Co., Ltd., Nanjing, China), respectively. Briefly, the MDA-MB-231 cells were seeded at a density of 3×10^5^ cells per well in 24-well culture plates, and treated as described previously. Cells were collected in an Eppendorf tube 24 h after irradiation and washed twice with PBS by centrifugation. The supernatants were discarded. To analyze the cell cycle, 50 μl RNase A and 200 μl PI were added to each tube, and the tube contents were mixed in the dark at room temperature for 20 min, followed by FCM testing. To detect apoptosis, 500 μl PBS, 5 μl Annexin V-FITC and 5 μl PI were added to each tube, and the contents of the tube were mixed in the dark at room temperature for 15 min, followed by FCM testing. Cell Quest software (Becton-Dickinson) was used to acquire and analyze the data, and the data are expressed as cell percentages.

### Statistical processing

The experimental data are expressed as mean ± standard deviation (SD). A one-way ANOVA test was used for statistical analysis using the statistical software SPSS 12.0 (SPSS, Inc., Chicago, IL, USA). P<0.05 was considered to indicate a statistically significant result.

## Results

### Expression of Smac protein in MDA-MB-231 cells

**Figure 2:**
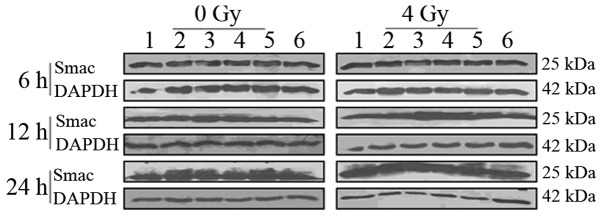
Expression of Smac protein in MDA-MB-231 cells at different times after 0- and 4-Gy X-ray radiation. 1, control; 2, CRAd.pEgr-1-Smac; 3, hypoxia; 4, CRAd.p; 5, CRAd.pEgr-1-Smac + H; 6, CRAd.p + H. Smac, second mitochondria-derived activator of caspase; GAPDH, Glyceraldehyde 3-phosphate dehydrogenase; CRAd, conditionally replicative adenovirus.

At 6, 12 and 24 h after sham irradiation (0 Gy), the difference in Smac protein expression between groups was small, which indicated that the Egr-1 promoter had no function in the absence of irradiation and was unable to express the characteristic of regulating the downstream gene (Fig. 2). At 6 h after 4-Gy irradiation, there was no significant increase in the level of Smac expression; while after 12 and 24 h, the Smac expression level was increased in the CRAd.pEgr-1-Smac and CRAd.pEgr-1-Smac + H groups, particularly in the latter.

### Changes in cell proliferation

**Figure 3:**
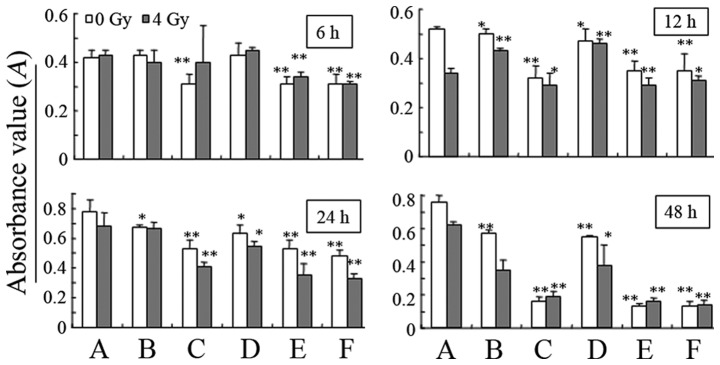
Absorbance value of MDA-MB-231 cells at different times after 0- and 4-Gy X-ray radiation. A, control; B, CRAd.pEgr-1-Smac; C, hypoxia; D, CRAd.p; E, CRAd.pEgr-1-Smac + H; F. CRAd.p + H. ^*^P<0.05 and ^**^P<0.01 vs. control. Smac, second mitochondria-derived activator of caspase; CRAd, conditionally replicative adenovirus.

As shown in Fig. 3, at 6 h after 0-Gy irradiation, hypoxia inhibited the growth of MDA-MB-231 cells; there was a statistically significant difference in cell proliferation between the hypoxia and normal control groups (P<0.01). At 12 h, the transfected virus also inhibited MDA-MB-231 cell growth, and the inhibition was time-dependent. At 6 h after 4-Gy irradiation, there was no significant reduction in the proliferation of the cells transfected with the CRAd.pEgr-1-Smac virus, while the proliferation was reduced significantly following hypoxia (P<0.01). At 12 h, the cell proliferation of each group decreased significantly (P<0.01), and at 48 h, it reached minimum values.

### Changes in MDA-MB-231 cell cycle progression

**Figure 4:**
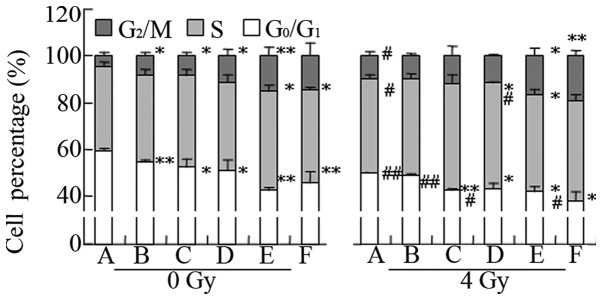
Change of MDA-MB-231 cell percentage in each cell cycle phase after 0- and 4-Gy X-ray radiation. A, control; B, CRAd.pEgr-1-Smac; C, hypoxia; D, CRAd.p; E, CRAd.pEgr-1-Smac + H; F, CRAd.p + H. ^*^P<0.05 and ^**^P<0.01 vs. control; ^#^P<0.05 and ^##^P<0.01 vs. 0 Gy. Smac, second mitochondria-derived activator of caspase; CRAd, conditionally replicative adenovirus.

Apoptosis occurs in various cell cycle phases, and cell cycle progression and apoptosis are closely associated. As shown in Fig. 4, the transfection with CRAd.pEgr-1-Smac or CRAd.p in combination with hypoxia may lead to an increase in the percentage of MDA-MB-231 cells in the S phase. The CRAd.pEgr-1-Smac + H and CRAd.p + H groups had a significantly increased percentage of cells in the S phase compared with the control group (P<0.01), and a significantly increased percentage of cells in the G_2_/M phase compared with the normal control group (P<0.05). The 4-Gy irradiation and hypoxia led to increases in the percentages of cells in the S and G_2_/M phases, while transfection with CRAd.pEgr-1-Smac did not change the cell cycle markedly. The 4-Gy irradiation of normal control cells increased the percentage of cells in the S and G_2_/M phases significantly compared with 0-Gy irradiation (P<0.05), while no significant changes occurred in the other groups.

### Changes in apoptosis of MDA-MB-231 cells

**Figure 5:**
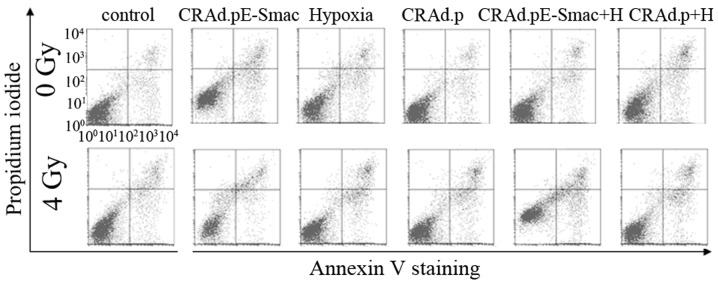
Flow cytometric detection of the apoptosis of MDA-MB-231 cells after 0- and 4-Gy X-ray radiation. Smac, second mitochondria-derived activator of caspase; CRAd, conditionally replicative adenovirus.

**Figure 6:**
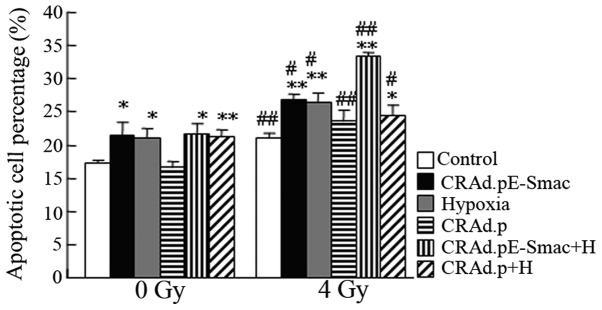
Change in the apoptotic percentage of MDA-MB-231 cells after 0- and 4-Gy X-ray radiation. ^*^P<0.05 and ^**^P<0.01 vs. control; ^#^P<0.05 ^##^P<0.01 vs. 0 Gy. Smac, second mitochondria-derived activator of caspase; CRAd, conditionally replicative adenovirus.

In Figs. 5 and 6, transfection with CRAd.pEgr-1-Smac and hypoxia following 0-Gy irradiation is shown to induce MDA-MB-231 cell apoptosis significantly when compared with the normal control (P<0.05); transfection with CRAd.p had no such effects, but when combined with hypoxia was able to induce cell apoptosis significantly (P<0.01). When treated with 4 Gy of radiation, the cell apoptosis events in each group were the same as those when 0 Gy of radiation was used. The CRAd.pEgr-1-Smac + H group presented the greatest increase in cell apoptosis. In addition, in this group, the percentage of cell apoptosis when 4 Gy of radiation was administered was significantly higher compared with that when 0 Gy of radiation was used (P<0.01).

## Discussion

The efficient expression of radiation-inducible therapeutic genes in tumor cells is crucial in gene-radiotherapy. Only by the overexpression of the gene with its corresponding functions may the combined effects of gene therapy and radiotherapy be achieved. CRAd, also called oncolytic adenovirus, is an ideal expression vector, which is able to replicate in tumor cells and produce a cascade effect (21,22). Relevant studies (5–7,23) have confirmed that ionizing radiation is able to induce the Egr-1 promoter to activate the expression of the downstream gene, which generates oxygen-free radicals acting on the six serum response element CArG. Hypoxia in solid tumors leads to the reduction of the free radicals induced by radiation, affecting the efficiency of the Egr-1 promoter.

The present study used the hypoxia-inducible characteristic of HRE to enhance the replication ability of CRAd under hypoxic conditions, and also strengthened the mediated efficiency of radiation on the Egr-1 promoter, increasing the expression of Smac, and thus realized dual activation by radiation and hypoxia.

At 6, 12 and 24 h after sham irradiation (0 Gy), the difference in Smac protein expression between the groups was small, indicating that the Egr-1 promoter had no function in the absence of irradiation, and was not able to regulate the downstream gene. The presence of Smac expression observed in each group may be due to endogenous expression in the cell, not the exogenous Smac expression. At 6 h after treatment with 4 Gy of radiation, there was no significant increase in Smac expression, while after 12 and 24 h, Smac expression was increased in the CRAd.pEgr-1-Smac group, and markedly increased in the CRAd.pEgr-1-Smac + H group. These results indicated that radiation activated the Egr-1 promoter. Hypoxia induced an increase in the replication of CRAd, suggesting that this experiment achieved the targeted overexpression of the therapeutic Smac gene with radiation and hypoxia.

At 12 h after 0-Gy irradiation, the transfected virus inhibited the MDA-MB-231 cell growth, and the inhibitory effect was time-dependent. This was likely due the CRAd itself being able to inhibit the tumor cell proliferation (24). At 6 h after 4-Gy irradiation, there was no significant reduction in the proliferation of the cells transfected by the CRAd.pEgr-1-Smac virus, while the proliferation reduced significantly following hypoxia (P<0.01). At 12 h, the cell proliferation of each group decreased significantly (P<0.01), and at 48 h, the proliferation reached minimum values. These results indicate that the expression of Smac protein was at a low level 6 h after irradiation and had no function. With the progression of time, the expression level increased and Smac was able to fully inhibit cell proliferation.

After exposure to ionizing radiation, most cells are in G2 arrest, ensuring the DNA damage is repaired and conducive to cell survival (25). Furthermore, G2 arrest is also associated with cell radiosensitivity and may increase cell radiosensitivity, which is useful in the radiation therapy of tumor cells (26). The changes in MDA-MB-231 cell cycle progression demonstrate that the conditions in the present study maintained the MDA-MB-231 cells in G_2_/M phase arrest, which may increase the cellular radiosensitivity and aid tumor radiotherapy.

The aim of tumor gene-radiotherapy includes not only the inhibition of cell proliferation, but also the induction of cell death. Apoptosis is one type of cell death. Ionizing radiation is the basis of clinical radiotherapy, which is able to induce tumor cell apoptosis directly and block the cell cycle checkpoint. Initiating apoptosis by using certain radiotherapy strategies may inhibit the growth of tumor cells effectively, being quite important to tumor radiotherapy. Smac is one type of apoptosis-inducing factor. An anti-tumor study conducted with Smac in recent years indicated that the overexpression of Smac may cause tumor cell apoptosis, and increase the sensitivity of tumor cells to chemotherapy (27). The increase in apoptosis of MDA-MB-231 cells with Smac overexpression suggests that when the cells transfected with CRAd.pEgr-1-Smac are in a hypoxic condition, treatment with ionizing radiation is able to induce the highest level of cell apoptosis. Therefore, the present study indicates that the maximum efficacy may be achieved by using the gene-radiotherapy strategy.

In conclusion, this study achieves the overexpression of Smac with hypoxia and radiation; its ability to inhibit cell proliferation and induce the apoptosis of MDA-MB-231 breast cancer cells is clearly apparent. The overexpression of Smac may also result in cell cycle arrest by enabling the G_2_/M phase, i.e. the radiation-sensitive cell cycle phase, which is also likely to facilitate the subsequent radiotherapy. This study explored a gene-radiotherapy strategy for treating breast cancer, and this strategy is expected to solve gene selection and targeting problems. First, the hypoxic microenvironment of solid tumors was used to overcome the ineffectiveness of gene-radiotherapy caused by hypoxia. Secondly, the CRAd vector was used to deliver a therapeutic gene to tumor cells subjected to increased radiation levels and a hypoxic status. Radiotherapy may be fully integrated with the favorable and unfavorable conditions of genes to maximize the benefits of radiotherapy. This study provides a new method for the clinical treatment of breast cancer.
